# Drinking Water Uranium and Potential Health Effects in the German Federal State of Bavaria

**DOI:** 10.3390/ijerph14080927

**Published:** 2017-08-18

**Authors:** Andre Banning, Mira Benfer

**Affiliations:** 1 Hydrogeology Department, Institute of Geology, Geophysics and Mineralogy, Ruhr-Universität Bochum, Universitätsstr. 150, 44801 Bochum, Germany; 2 Soil Science and Soil Ecology Department, Institute of Geography, Ruhr-Universität Bochum, Universitätsstr. 150, 44801 Bochum, Germany; mira.benfer@rub.de

**Keywords:** uranium, public health, odds ratio, relative risk, Bavaria, disease, cancer, liver, thyroid, groundwater

## Abstract

Mainly due to its nephrotoxic and osteotoxic potential, uranium (U) increasingly finds itself in the spotlight of environmental and health-related research. Germany decided on a binding U guideline value in drinking water of 10 µg/L, valid since 2011. It is yet widely unknown if and how public health was affected by elevated U concentrations before that. In this ecological study we summarized available drinking water U data for the German federal state of Bavaria (703 analyses in total for 553 different municipalities) at county level (for 76 out of 96 Bavarian counties, representing about 83% of Bavaria’s and about 13% of Germany’s total population) in terms of mean and maximum U concentration. Bavaria is known to regionally exhibit mainly geogenically elevated groundwater U with a maximum value of 40 µg/L in the database used here. Public health data were obtained from federal statistical authorities at county resolution. These included incidence rates of diagnosed diseases suspected to be potentially associated with chronic U uptake, e.g., diseases of the skeleton, the liver or the thyroid as well as tumor and genito-urinary diseases. The datasets were analyzed for interrelations and mutual spatial occurrence using statistical approaches and GIS as well as odds ratios and relative risks calculations. Weak but significant positive associations between maximum U concentrations and aggregated ICD-10 diagnose groups for growths/tumors as well as liver diseases were observed, elevated incidence rates of thyroid diseases seem to occur where mean drinking water U concentrations exceed 2 µg/L. Here, we discuss obtained results and their implications for potential impacts of hydrochemistry on public health in southeast Germany.

## 1. Introduction

In recent years, uranium (U) has become a broadly studied heavy metal in the fields of environmental and health-related research. It is a natural constituent of the earth’s crust and occurs in all of its rocks, soils and fluids. Uranium mobility and distribution in the environment are governed by its Eh- and pH-dependent speciation with U(VI) being the mobile form (reduced U(IV) tends to be immobilized as e.g., the mineral phase uraninite UO_2_), especially in bicarbonate-containing waters where stable U(VI)-carbonato-complexes are dominant [1,2,3]. Large-scale U distribution is controlled by geology, mainly due to the heavy metal’s incompatible behavior in magmatic differentiation which leads to elevated U contents in felsic magmatites like granites and pegmatites, and associated sediment basins and finally groundwater. Intermediate U sinks and sources include organic-rich sediments like fen peats and gleyic soils [4,5,6,7] reflecting the element’s affinity towards organic matter sorption. Inorganic phosphorus fertilizers, former U mining sites, depleted U ammunition, emissions from the nuclear industry as well as combustion of coal and other fuels represent potential anthropogenic U sources [8,9,10,11,12,13].

Uranium is a heavy metal with both radiotoxic and chemotoxic properties potentially leading to adverse health effects in exposed populations whereby not only total concentration but also U speciation/complexation are decisive (e.g., [14,15]). The main U exposure pathway appears to be uptake via drinking water in most cases indicating that populations supplied with drinking water from U-rich groundwater resources may exhibit elevated health risks (e.g., [16]) associated with the nephrotoxic potential of ingested U mainly as it damages the kidney tubular cells [17]. Beside this geologically caused intake differences via drinking water/soft beverages, food represents an additional U exposure pathway whereby vegetable foodstuff delivers a slightly higher percentage of the daily U ingestion than animal products for an average omnivore diet [18]. Moreover, the skeleton is another target for ingested U absorption in the body. It can affect bone development and maintenance, especially in young individuals. The accumulation mechanism is mainly the substitution of U for calcium in the osseous tissue [19]. Most U is excreted rapidly from the body via urine and feces, but about 1−1.5% is assumed to be adsorbed in the gastrointestinal tract in adults. In bones, U retention has a half-live between 70 and 200 days, 80−90% of deposited U leaves the body after 1.5 years [17,19,20]. As an alpha particle emitter, adsorbed radioactive U may also lead to DNA damage and therefore carcinogenesis. Wagner et al. [21] found in an ecological study in the U.S. that incidences of several cancer types including kidney cancer may be enhanced in areas supplied by groundwater with elevated U concentrations. Earlier studies suggested associations between elevated groundwater U daughter radionuclide (radium, radon) concentrations with bone, lung, breast and blood cancer [22,23,24,25]. On the other hand, several Finnish studies [26,27,28] did not find adverse health effects associated to elevated drinking water U. In answer to these findings, health authorities implemented drinking water guideline values for U which partly have changed over time. WHO increased its provisional U guideline value from 15 to 30 µg/L [11] as a reaction to the findings of [28]. In Germany, authorities for the first time established a fixed U threshold value of 10 µg/L, valid since 2011 [29].

This study examines potential associations between elevated groundwater U concentrations and public health effects in the German federal state of Bavaria using publicly available hydrochemical data and the spatial distribution of different diseases incidence data provided by German statistical authorities. The aim is to test the hypothesis that elevated groundwater U is associated with elevated incidence rates of diseases of the genito-urinary system, the skeleton, the liver or the thyroid as well as tumor diseases. Occurrence of high groundwater U is documented in South-East German Bavaria, elevated concentrations are widespread in the northern part of the state (Franconia). Uranium derives from the weathering of uraniferous apatite concretions in Triassic aquifer sediments [30]. While Bavaria’s southern part is generally less affected, localized cases of high groundwater U are mainly associated with mobilization from organic-rich bog areas, partly triggered by agricultural activity [7]. Using different datasets and statistical approaches for some parts of Bavaria, Radespiel-Tröger and Meyer [31] found significantly increased risks of leukemia for men, and kidney and lung cancer for women when U exposure was elevated. Bavaria produces the majority of its drinking water (86%) from groundwater [32] (data from 2013).

## 2. Materials and Methods

### 2.1. Hydrochemical Data

Drinking water U concentrations were obtained from a freely available internet data resource provided by the German non-profit organisation “foodwatch” which collected and published U concentration data for public water supply systems from German health and environmental authorities obtained between 2000 and 2009. For Bavaria, this dataset includes 703 single U concentration values from 553 municipalities [33]. The dataset also reflects U distribution in Bavarian groundwater, the by far most important drinking water source, because of the special highly decentralized drinking water supply system consisting of about 2350 municipal water suppliers in the federal state. Analytical detection limits were between 0.1 and 0.5 µg/L (2 µg/L at one single location). For Bavarian groundwater, federal authorities report U concentrations >10 µg/L in 7.5% out of 1566 analyzed samples with a maximum value of 57 µg/L [34].

### 2.2. Diseases Incidence Rates Data

Numbers on diagnosed cases of selected diseases suspected to be associated with elevated U exposure as well as population numbers on county level for 2014 were supplied by federal statistical authorities (Bayerisches Landesamt für Statistik) in summer 2016. Diagnoses originated from hospitals and were registered for the places of residents of the patients. Information on potential differences in diagnosing or registration practices between counties and federal states were not documented, and are therefore not considered in this study. Population data reports about 10.5 million people in the studied 76 (out of a total of 96) counties in Bavaria representing about 83% of the total Bavarian population and 13% of Germany’s 81.2 million inhabitants in total. The following ICD-10 coded groups of diseases were considered in this study: C00-D48 (malignant, benign and other growths/tumors), E00-E07 (diseases of the thyroid), K70-K77 (diseases of the liver), M80-M99 (osteopathy, chondropathia and other diseases of the musculoskeletal system), N00-N99 (diseases of the genito-urinary system), Q00-Q99 (congenital malformation, deformities and chromosome anomalies). Incidence rates for these ICD codes were also calculated for the overall German population based on publicly available data from [35] for 2014 as reference values. Health data 5−11 years younger than U exposure data was used because latency periods are expected to last 10 years to several decades, as concluded from earlier epidemiological studies on U [36,37].

### 2.3. Data Processing and Statistical Approaches

Where several U analyses were available for the same location, arithmetic mean values and maximum values were considered for the applied statistical approaches. For data below analytical detection limits, the value 0 was assumed. Available U data was georeferenced using the program ArcGIS 10.1 (Esri, Redlands, CA, USA) by assigning spatial coordinates to the tabulated data which only offered municipality names. In some cases, only waterworks names were offered, these were assigned to the respective municipality. Municipalities were then allocated to the different administrative counties. Uranium data was spatially adapted to county borders to be comparable to diseases diagnose data. For each county, a mean U concentration was calculated from all allocated municipality U values, and used for further statistical calculations. Additionally, the maximum U value in each county was used for comparison. Counties without available U analyses in the database (*n* = 20) were not considered leaving a dataset of *n* = 76 counties, 7 of which (9%) were represented by a single U analysis (cf. Table S1 in Supplementary Materials). For the medical data, incidence rate values were calculated for every ICD code group for each county. A total of 461,456 individual cases were included for Bavaria, patients diagnosed in Bavaria but living outside of the federal state (about 5% of all cases) were not included. 

The program SPSS (IBM, Armonk, NY, USA) was used to calculate Pearson correlation coefficients to evaluate potential statistical associations between drinking water U concentrations and incidence rates for the different ICD code groups. Odds ratios (OR) and relative risks (RR) were calculated to compare the probabilities of county disease incidence rates to be higher than the national average with and without the population being exposed to a certain level of drinking water U in a given county (risk factor). The U level was set for two different cases—counties with a maximum U drinking water concentration of >10 µg/L, and those with a mean U concentration >2 µg/L. The latter is due to a guideline given by German authorities for bottled water used for the preparation of baby food [16]. It also corresponds to the original WHO guideline for drinking water before it was increased stepwise to the current value of 30 µg/L [11]. ICD incidence rates calculated for the overall German population served as reference values. This approach uses a two-by-two frequency table (Table 1).

The following equation was then used for OR calculation: OR = (a/c)/(b/d) = ad/bc. An odds ratio of 1 means that there is no difference in incidence rates between conditions with and without the risk factor. OR > 1 suggests an elevated disease incidence rate (on county level) when the risk factor applies, OR < 1 a lower one.

The relative risk (RR), also referred to as risk ratio, was calculated as the quotient of incidence rates with and without risk factor, i.e., RR = a/(a + c)/(b/(b + d)). RR expresses the difference in disease risk between counties with and without the risk factor (mean drinking water U > 2 µg/L or max. drinking water U > 10 µg/L) with values >1 suggesting an elevated risk at higher U exposure. 

95% confidence intervals for OR and RR were calculated after [38].

In epidemiological research, odds ratios are commonly used in case-control studies while relative risks are often calculated in cohort studies or randomized controlled trials. In this study, both were calculated for the sake of comparison (also with literature data like [31]), and because our approach cannot clearly be assigned to one of the mentioned study types. 

Using GIS, maps were created to visualize spatial associations between drinking water U and incidences of the studied ICD-10 coded diseases.

## 3. Results

The number of diagnosed cases and calculated incidence rates (2014) for the selected ICD-10 coded groups of diseases for the total German population (81.2 million people) are shown in Table 2.

Table S1 in the Supplementary Materials presents U concentration data in drinking water as well as cases and calculated incidence rates (2014) for the selected ICD-10 coded groups of diseases for the studied German federal state (data shown by counties). Furthermore, it gives an overview of the number of municipalities and available U analyses per county.

For Bavaria, the mean U concentration exceeds 2 µg/L in 30 counties (39% of the considered 76 counties), 10 counties (13%) exhibit maximum U values >10 µg/L in this dataset, mainly in the northern part of the federal state. The overall maximum concentration is 39.9 µg/L. Note that only one U value was available for some counties (cf. Table S1 in Supplementary Materials). Incidence rates of the ICD-10 coded groups of diseases C00-D48, E00-E07, K70-K77, M80-M99, N00-N99 and Q00-Q99 exceed the overall German average in 17%, 55%, 26%, 47%, 28% and 18% of the counties, respectively.

Table 3 reports Pearson correlation coefficients for mean and maximum U concentrations per county with incidence rates.

**Figure 1:**
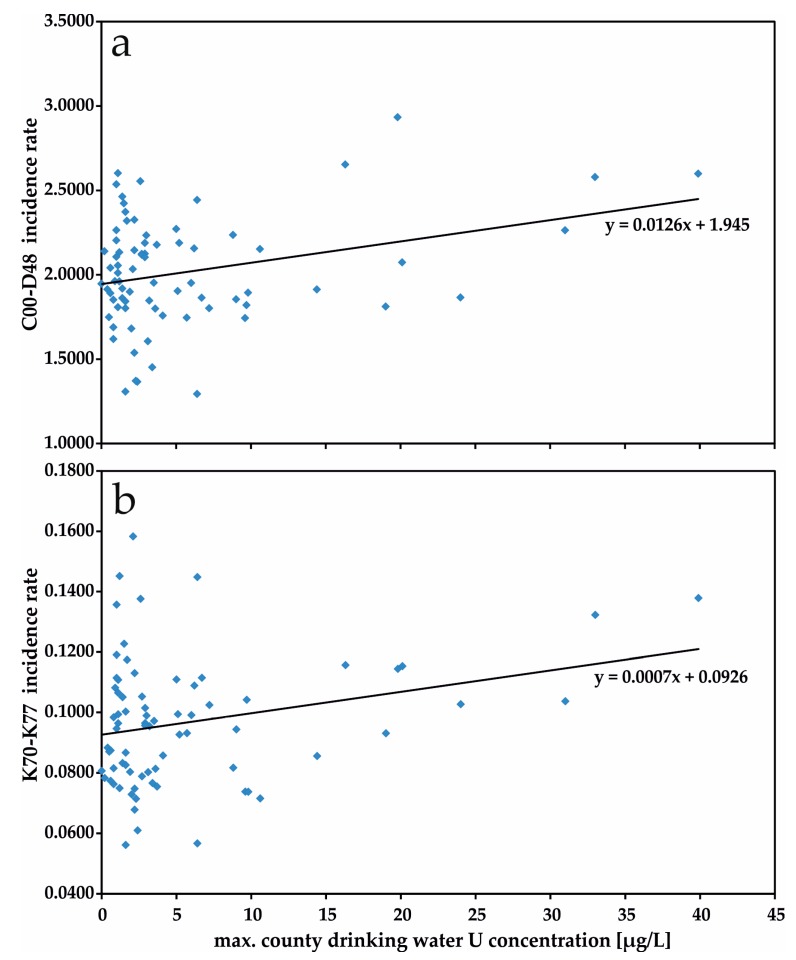
Bivariate correlations of maximum U concentrations in drinking water and diseases groups incidence rates. (**a**) C00-D48; (**b**) K70-K77.

Statistically significant positive Pearson correlation (*p* < 0.05) was observed for Bavarian drinking water U concentrations (mean and maximum values) with ICD-10 code group K70-K77 (liver diseases), and for maximum U values with ICD-10 code group C00-D48 (growths/tumors; *p* < 0.01). Figure 1 visualizes bivariate correlations between maximum U concentrations in drinking water and those ICD coded groups of diseases incidence rates with detected significant Pearson correlation.

Results of odds ratio (OR) and relative risk (RR) calculations for the risk factor “mean drinking water U concentration >2 µg/L” are presented in Table 4.

Table 5 shows OR and RR results for the risk factor “maximum drinking water U concentration >10 µg/L” for the federal state.

OR and RR show statistically insignificant values <1 or close to unity for M80-M99, N00-N99 and Q00-Q99 for both applied risk factors, i.e., nearly indifferent incidence numbers in the considered groups of musculoskeletal and genito-urinary diseases as well as malformations. For the growths/tumors (C00-D48) and the liver diseases (K70-K77) groups, slightly elevated (but statistically insignificant) values >1 were calculated for the “mean drinking water U > 2 µg/L” risk factor, but statistically significant ORs of 4.22 and 3.40, respectively, when the occurrence of maximum concentrations >10 µg/L is taken into account. Calculated significant RRs (2.93 and 2.20) exhibit a similar pattern for these groups. Inversely, the ICD-10 group E00-E07 (thyroid diseases) has significant OR and RR > 1 for the mean >2 µg/L risk factor (significant on the 95% level), but statistically insignificant values >1 for the max. >10 µg/L risk factor. 

**Figure 2:**
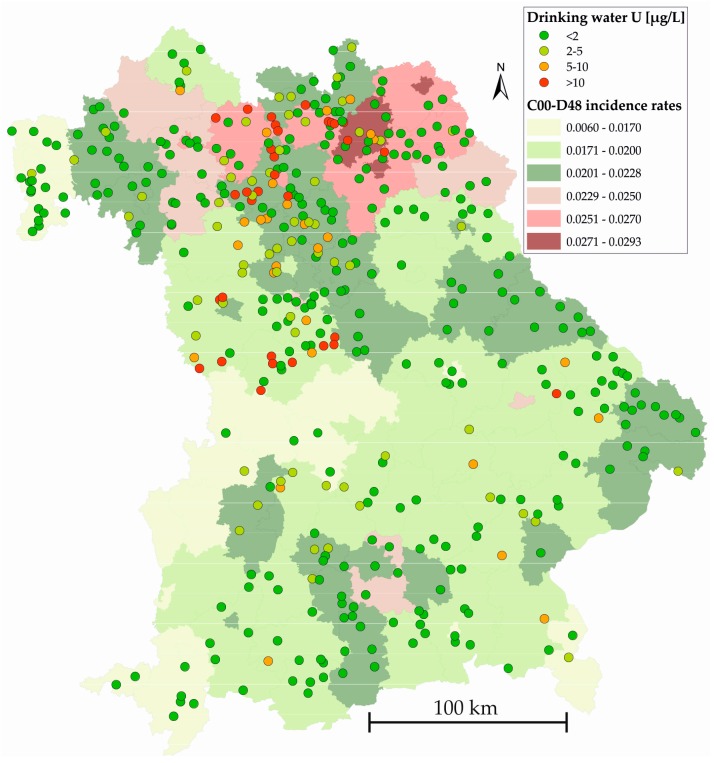
Distribution of U drinking water concentrations and C00-D48 incidence rates in Bavaria. Greenish colors mark counties below, reddish colors above the national average of 0.0228.

Figure 2 presents a map of Bavaria indicating the spatial correspondence of drinking water U concentrations and incidence rates of C00-D48.

The Bavarian distribution of drinking water U concentrations (Figure 2) reflects the aforementioned tendency to higher values in the northern part of the federal state due to geological reasons (cf. Chapter 1). Two areas, one in the northernmost part, one in the central north, are especially affected by elevated concentrations. Higher C00-D48 incidence rates also appear to occur predominantly in the northern counties of Bavaria. Although there is definitely no striking spatial correlation between these two parameters, a general north-south gradient appears to exist for both. The same is generally true for the ICD-10 coded group of liver diseases (K70-K77, not shown) while there is no spatial coincidence between U concentrations and the studied group of thyroid diseases (E00-E07, not shown).

## 4. Discussion

In this study, we found weak but statistically significant positive correlations between drinking water U concentrations and incidence rates of several groups of diseases for Bavaria: C00-D48 (malignant, benign and other growths/tumors) and K70-K77 (diseases of the liver). These groups, together with E00-E07 (diseases of the thyroid) also exhibited highest calculated values for odds ratios and relative risks. Both are elevated and statistically significant for C00-D48 and K70-K77 when occurrence of a maximum U concentration >10 µg/L in counties of residence is considered as the risk factor, but not if mean concentrations >2 µg/L U are used. Results—though no differentiation between types of cancer/growths was considered here—support the findings of [31] who found slightly increased risks of leukemia, kidney cancer and lung cancer in Bavarian municipalities associated with increased drinking water U concentrations. These authors documented elevated relative risks for intermediate (>1 µg/L; RR = 1.12) and high (>5 µg/L; RR = 1.28) U exposure. There also seems to be a common spatial tendency for both drinking water U and undifferentiated tumor/growth incidence rates with higher values in the northern part of the federal state. Relatively little is known about adverse effects of U exposure on the liver [39] report that beside kidney and skeleton, the liver is a third target organ for U uptake in the human body hosting 16% of the normal adult body burden of U (90 µg). Existing animal studies provide evidence that U exposure can damage the liver, and human liver dysfunction was observed upon acute uptake of a high U dose. However, the etiology of both effects is yet unknown [39].

The thyroid diseases group (E00-E07) showed no statistically significant correlation, but significantly elevated OR/RR when mean U concentration >2 µg/L was applied as the risk factor. A spatial pattern, however, was not detected. It is known that radionuclides, especially those emitted after nuclear disasters, can substantially increase the risk for thyroid cancer since the thyroid gland is highly sensitive to the carcinogenic effects of radiation [40]. It remains unclear if observations from the present study are associated with the effect of U radiotoxicity on the thyroid, future research might address this question. Neither significant statistical associations nor correlating spatial patterns between drinking water U concentrations and incidence rates of M80-M99 (osteopathy, chondropathia and other diseases of the musculoskeletal system), N00-N99 (diseases of the genito-urinary system) and Q00-Q99 (malformation, deformities and chromosome anomalies) were found.

Results obtained in this study must at least partly be considered a look into the past because U concentration data from earlier than 2011 was used. Drinking water U concentrations will partly have decreased since then, mainly because of remediation measures taken by water suppliers as a reaction towards the newly implemented guideline value. Further limitations of this study include: the limited spatial resolution of the used diseases incidence data; the fact that only groups of diseases, not single diagnoses were considered; the fact that no potentially important parameters save U concentration in drinking water were considered (age, diet, sex, socio-economic status, habits like smoking, adipositas, individual use of tap water, general tap water composition, U intake from food and bottled water etc.); the relatively inhomogeneous spatial distribution of the used U concentration data; the fact that mean U values had to be calculated for single locations, and again for single counties (though maximum values were also considered); precision and analytical detection limits of the used U data, U speciation/complexation, ecologic design of the study. These limitations should be considered in the interpretation of obtained results, and may be addressed in future ecological research. 

## 5. Conclusions 

In this study, we collected and analyzed data from the German federal state of Bavaria on drinking water U concentrations and incidence rates of selected groups of diseases potentially associated with elevated U exposure (tumors/growths, liver, thyroid, musculoskeletal and genito-urinary diseases, and congenital malformations). Pearson correlation, odds ratios/relative risk calculation and GIS visualization were used to study potential associations between these parameters.

Areas of drinking water U exceeding the German guideline value of 10 µg/L are documented for the federal state with a north-south gradient in Bavaria. We found weak but significant positive correlations between drinking water U concentrations and incidence rates of tumors/growths and liver diseases. Odds ratios and relative risks for these disease groups showed significantly elevated values for counties with maximum U drinking water concentrations >10 µg/L, for thyroid diseases only for counties with mean drinking water U >2 µg/L. The mentioned north-south gradient of U concentrations was roughly reproduced by the distribution of tumors/growths and liver diseases incidence rates. No statistical or spatial correlations were found for genito-urinary and musculoskeletal diseases as well as for congenital malformations. 

Our results support an earlier study in Bavaria describing elevated risks for some types of cancer at high drinking water U exposure. Furthermore, especially the rather unexpected finding that incidence rates and OR/RR values for liver and thyroid diseases appear to coincide with elevated drinking water U warrants further research. Future studies should take the temporal development of incidence rates as a potential response to implemented drinking water guidelines into account. 

## Figures and Tables

**Table 1:** Odds ratios calculation scheme.

No. of Counties	With Risk Factor ^3^	Without Risk Factor ^4^
with high incidence ^1^	a	b
with low incidence ^2^	c	d

^1^ incidence rates above the overall German average; ^2^ incidence rates below the overall German average; ^3^ mean drinking water U > 2 µg/L (resp. max. U > 10 µg/L); ^4^ mean drinking water U < 2 µg/L (resp. max. U < 10 µg/L).

**Table 2:** Cases and incidence rates in total German population (81.2 million people) 2014.

ICD-10 Code	Explanation	New Cases	Incidence Rate	Incidence Rate/100,000
C00-D48	growths/tumors	1,852,202	0.0228	2281
E00-E07	thyroid diseases	98,166	0.0012	121
K70-K77	liver diseases	87,509	0.0011	108
M80-M99	musculoskeletal diseases	132,771	0.0016	164
N00-N99	genito-urinary diseases	1,044,701	0.0129	1287
Q00-Q99	malformation, deformities and chromosome anomalies	104,793	0.0013	129

**Table 3:** Pearson correlation coefficients for mean and maximum U concentrations per county with incidence rates (*n* = 76).

	C00-D48	E00-E07	K70-K77	M80-M99	N00-N99	Q00-Q99
mean U	0.220	0.084	0.242	0.057	0.048	0.055
*p*	0.06	0.47	0.04 ^1^	0.63	0.68	0.64
max. U	0.302	0.062	0.264	0.064	0.062	0.026
*p*	0.008 ^2^	0.60	0.02 ^1^	0.58	0.60	0.83

^1^ *p* < 0.05; ^2^ *p* < 0.01.

**Table 4:** Odds ratio (OR) and relative risk (RR) calculations using mean drinking water U concentration >2 µg/L as risk factor. 95% CI: 95% confidence intervals (lower and upper limit).

Bavaria	C00-D48	E00-E07	K70-K77	M80-M99	N00-N99	Q00-Q99
OR	1.31	3.22	1.78	0.67	1.17	1.15
CI (OR)	0.40; 4.22	1.18; 8.97	0.66; 4.82	0.26; 1.69	0.43; 3.22	0.36; 3.67
RR	1.24	1.56	1.51	0.80	1.12	1.12
CI (RR)	0.48; 3.23	1.11; 2.17	0.76; 2.99	0.47; 1.37	0.55; 2.29	0.44; 2.85

**Table 5:** Odds ratio (OR) and relative risk (RR) calculations using maximum drinking water U concentration >10 µg/L as risk factor. 95% CI: 95% confidence intervals (lower and upper limit).

Bavaria	C00-D48	E00-E07	K70-K77	M80-M99	N00-N99	Q00-Q99
OR	4.22	2.07	3.40	0.43	1.14	0.45
CI (OR)	1.15; 15.5	0.57; 7.51	1.00; 11.6	0.12; 1.56	0.31; 4.23	0.07; 3.13
RR	2.93	1.89	2.20	0.60	1.10	0.51
CI (RR)	1.23; 7.02	0.60; 5.93	1.11; 4.36	0.25; 1.44	0.43; 2.76	0.09; 2.85

## Supplementary Materials

The following are available online at www.mdpi.com/1660-4601/14/8/927/s1, Table S1: Drinking water U concentrations [μg/L] and incidence rates 2014 in 76 counties of Bavaria (10.5 million people in total) used in this study.
