# Supplementary material for: Drinking Water Uranium and Potential Health Effects in the German Federal State of Bavaria
Source: Int J Environ Res Public Health. 2017 Aug 18;14(8):927. doi: 10.3390/ijerph14080927 (PMC5580629; doi:10.3390/ijerph14080927)
Supplement: Supplementary file 1 [file ijerph-14-00927-s001.pdf]

**Table S1.** Drinking water U concentrations [µg/L] and incidence rates 2014 in 76 counties of Bavaria (10.5 million people in total) used in this study.

| County | mun.[1] | U anal.[2] | mean U | max. U | C00-D48 | E00-E07 | K70-K77 | M80-M99 | N00-N99 | Q00-Q99 |
|---|---|---|---|---|---|---|---|---|---|---|
| Aichach-Friedberg | 24 | 2 | 6.00 | 9.00 | 0.0185 | 0.0014 | 0.0009 | 0.0014 | 0.0103 | 0.0013 |
| Amberg-Sulzbach | 27 | 5 | 0.14 | 0.40 | 0.0191 | 0.0013 | 0.0009 | 0.0015 | 0.0115 | 0.0013 |
| Ansbach | 58 | 29 | 5.92 | 19.0 | 0.0181 | 0.0018 | 0.0009 | 0.0015 | 0.0104 | 0.0010 |
| Aschaffenburg | 32 | 4 | 0.45 | 1.60 | 0.0131 | 0.0002 | 0.0006 | 0.0010 | 0.0096 | 0.0006 |
| Augsburg | 46 | 4 | 1.35 | 2.90 | 0.0212 | 0.0013 | 0.0010 | 0.0017 | 0.0115 | 0.0013 |
| Bad Kissingen | 26 | 3 | 2.93 | 6.40 | 0.0244 | 0.0011 | 0.0014 | 0.0017 | 0.0120 | 0.0011 |
| Bad Tölz-W´hausen | 21 | 11 | 0.93 | 1.20 | 0.0213 | 0.0012 | 0.0015 | 0.0019 | 0.0105 | 0.0010 |
| Bamberg | 36 | 36 | 7.48 | 31.0 | 0.0226 | 0.0014 | 0.0010 | 0.0016 | 0.0105 | 0.0012 |
| Bayreuth | 33 | 15 | 1.45 | 16.3 | 0.0265 | 0.0012 | 0.0012 | 0.0015 | 0.0117 | 0.0010 |
| Berchtesgadener Land | 15 | 2 | 1.20 | 2.20 | 0.0154 | 0.0010 | 0.0007 | 0.0020 | 0.0112 | 0.0009 |
| Cham | 39 | 11 | 0.05 | 0.60 | 0.0204 | 0.0018 | 0.0009 | 0.0018 | 0.0132 | 0.0010 |
| Coburg | 17 | 17 | 8.97 | 20.1 | 0.0207 | 0.0013 | 0.0012 | 0.0016 | 0.0123 | 0.0011 |
| Coburg (City) | 1* | 1 | 2.10 | 2.10 | 0.0203 | 0.0016 | 0.0016 | 0.0015 | 0.0124 | 0.0012 |
| Dachau | 17 | 1 | 3.60 | 3.60 | 0.0180 | 0.0015 | 0.0008 | 0.0013 | 0.0105 | 0.0016 |
| Deggendorf | 26 | 7 | 0.73 | 5.10 | 0.0190 | 0.0016 | 0.0010 | 0.0014 | 0.0125 | 0.0011 |
| Dillingen a. d. Donau | 27 | 1 | 2.40 | 2.40 | 0.0137 | 0.0012 | 0.0006 | 0.0015 | 0.0102 | 0.0010 |
| Dingolfing-Landau | 15 | 4 | 1.18 | 1.60 | 0.0180 | 0.0015 | 0.0009 | 0.0017 | 0.0132 | 0.0012 |
| Ebersberg | 21 | 2 | 1.05 | 1.10 | 0.0201 | 0.0014 | 0.0010 | 0.0016 | 0.0106 | 0.0012 |
| Eichstätt | 30 | 1 | 0.80 | 0.80 | 0.0162 | 0.0009 | 0.0008 | 0.0016 | 0.0114 | 0.0012 |
| Erding | 26 | 5 | 1.80 | 4.10 | 0.0176 | 0.0010 | 0.0009 | 0.0014 | 0.0125 | 0.0016 |
| Erlangen-Höchstadt | 25 | 18 | 4.52 | 10.6 | 0.0215 | 0.0014 | 0.0007 | 0.0013 | 0.0109 | 0.0012 |
| Forchheim | 29 | 19 | 1.68 | 6.20 | 0.0216 | 0.0015 | 0.0011 | 0.0014 | 0.0108 | 0.0013 |
| Freising | 24 | 5 | 1.00 | 1.40 | 0.0186 | 0.0012 | 0.0011 | 0.0014 | 0.0108 | 0.0012 |
| Freyung-Grafenau | 25 | 8 | 0.03 | 0.20 | 0.0214 | 0.0014 | 0.0008 | 0.0014 | 0.0137 | 0.0011 |
| Fürstenfeldbruck | 23 | 6 | 1.80 | 3.00 | 0.0223 | 0.0010 | 0.0010 | 0.0018 | 0.0124 | 0.0012 |
| Fürth | 14 | 8 | 4.51 | 8.80 | 0.0224 | 0.0015 | 0.0008 | 0.0015 | 0.0129 | 0.0010 |
| Garmisch-P´kirchen | 22 | 6 | 0.88 | 1.90 | 0.0190 | 0.0010 | 0.0008 | 0.0026 | 0.0115 | 0.0011 |
| Günzburg | 34 | 2 | 1.50 | 2.30 | 0.0137 | 0.0009 | 0.0007 | 0.0012 | 0.0102 | 0.0008 |
| Haßberge | 26 | 21 | 10.2 | 39.9 | 0.0260 | 0.0014 | 0.0014 | 0.0019 | 0.0118 | 0.0013 |
| Hof | 27 | 7 | 0.20 | 1.10 | 0.0260 | 0.0010 | 0.0011 | 0.0018 | 0.0146 | 0.0007 |
| Ingolstadt | 1* | 2 | 0.45 | 0.50 | 0.0175 | 0.0011 | 0.0009 | 0.0015 | 0.0120 | 0.0010 |
| Kaufbeuren | 1* | 2 | 0.95 | 1.00 | 0.0220 | 0.0011 | 0.0012 | 0.0022 | 0.0145 | 0.0012 |
| Kelheim | 24 | 1 | 0.90 | 0.90 | 0.0196 | 0.0015 | 0.0011 | 0.0015 | 0.0122 | 0.0011 |
| Kitzingen | 31 | 16 | 0.83 | 2.20 | 0.0233 | 0.0010 | 0.0011 | 0.0019 | 0.0124 | 0.0009 |
| Kronach | 18 | 13 | 1.98 | 5.20 | 0.0219 | 0.0015 | 0.0009 | 0.0013 | 0.0131 | 0.0010 |
| Kulmbach | 22 | 15 | 5.48 | 19.8 | 0.0293 | 0.0012 | 0.0011 | 0.0015 | 0.0190 | 0.0010 |
| Landsberg am Lech | 31 | 5 | 0.68 | 1.20 | 0.0196 | 0.0012 | 0.0007 | 0.0016 | 0.0100 | 0.0009 |
| Landshut | 35 | 8 | 2.90 | 9.60 | 0.0174 | 0.0019 | 0.0007 | 0.0017 | 0.0118 | 0.0017 |
| Lichtenfels | 11 | 25 | 11.3 | 33.0 | 0.0258 | 0.0014 | 0.0013 | 0.0021 | 0.0132 | 0.0011 |
| Lindau | 19 | 4 | 1.98 | 6.40 | 0.0129 | 0.0004 | 0.0006 | 0.0010 | 0.0090 | 0.0006 |
| Main Spessart | 40 | 32 | 0.81 | 3.70 | 0.0218 | 0.0009 | 0.0008 | 0.0014 | 0.0110 | 0.0013 |
| Miesbach | 17 | 5 | 1.64 | 3.50 | 0.0195 | 0.0013 | 0.0010 | 0.0014 | 0.0093 | 0.0007 |
| Miltenberg | 32 | 20 | 0.72 | 3.40 | 0.0145 | 0.0003 | 0.0008 | 0.0011 | 0.0112 | 0.0005 |
| Mühldorf am Inn | 31 | 7 | 1.56 | 7.20 | 0.0180 | 0.0013 | 0.0010 | 0.0017 | 0.0138 | 0.0015 |
| München | 29 | 7 | 1.03 | 1.40 | 0.0246 | 0.0013 | 0.0008 | 0.0014 | 0.0108 | 0.0011 |
| München (City) | 1* | 2 | 1.00 | 1.10 | 0.0206 | 0.0012 | 0.0011 | 0.0014 | 0.0100 | 0.0011 |
| Neuburg-Sch´hausen | 18 | 6 | 1.07 | 3.10 | 0.0161 | 0.0011 | 0.0008 | 0.0017 | 0.0113 | 0.0009 |
| Neumarkt (Oberpfalz) | 19 | 8 | 0.36 | 1.00 | 0.0211 | 0.0014 | 0.0009 | 0.0015 | 0.0131 | 0.0015 |
| Neustadt-B. W´heim | 38 | 11 | 2.17 | 6.70 | 0.0186 | 0.0013 | 0.0011 | 0.0017 | 0.0145 | 0.0010 |
| Neustadt (Waldnaab) | 38 | 13 | 0.19 | 1.40 | 0.0192 | 0.0028 | 0.0011 | 0.0017 | 0.0119 | 0.0011 |
| Nürnberger Land | 27 | 30 | 1.51 | 5.00 | 0.0227 | 0.0017 | 0.0011 | 0.0017 | 0.0118 | 0.0010 |
| Oberallgäu | 28 | 9 | 0.82 | 2.00 | 0.0168 | 0.0008 | 0.0007 | 0.0016 | 0.0096 | 0.0008 |
| Ostallgäu | 45 | 9 | 0.72 | 1.10 | 0.0181 | 0.0011 | 0.0010 | 0.0018 | 0.0105 | 0.0012 |
| Passau | 38 | 4 | 0.85 | 2.70 | 0.0212 | 0.0013 | 0.0011 | 0.0017 | 0.0125 | 0.0008 |
| Pfaffenhofen (Ilm) | 19 | 4 | 2.28 | 3.20 | 0.0185 | 0.0012 | 0.0010 | 0.0014 | 0.0122 | 0.0011 |
| Regen | 24 | 18 | 0.08 | 0.80 | 0.0185 | 0.0010 | 0.0010 | 0.0017 | 0.0158 | 0.0009 |
| Regensburg | 41 | 12 | 1.48 | 9.80 | 0.0189 | 0.0017 | 0.0007 | 0.0014 | 0.0100 | 0.0011 |
| Regensburg (City) | 1* | 5 | 0.76 | 0.80 | 0.0169 | 0.0013 | 0.0008 | 0.0012 | 0.0105 | 0.0011 |
| Rhön Grabfeld | 37 | 9 | 1.91 | 9.70 | 0.0182 | 0.0007 | 0.0010 | 0.0016 | 0.0081 | 0.0009 |

| | | | | | | | | | | |
|---|---|---|---|---|---|---|---|---|---|---|
| Rosenheim | 46 | 7 | 1.01 | 1.60 | 0.0184 | 0.0014 | 0.0008 | 0.0016 | 0.0098 | 0.0014 |
| Roth | 16 | 29 | 2.59 | 14.4 | 0.0191 | 0.0014 | 0.0009 | 0.0016 | 0.0119 | 0.0012 |
| Rottal-Inn | 31 | 14 | 1.76 | 5.70 | 0.0175 | 0.0017 | 0.0009 | 0.0018 | 0.0142 | 0.0010 |
| Schwabach | 1* | 4 | 1.30 | 1.50 | 0.0242 | 0.0020 | 0.0012 | 0.0017 | 0.0128 | 0.0010 |
| Schwandorf | 33 | 13 | 0.11 | 1.00 | 0.0226 | 0.0019 | 0.0011 | 0.0017 | 0.0139 | 0.0012 |
| Schweinfurt | 29 | 12 | 1.06 | 1.60 | 0.0237 | 0.0009 | 0.0010 | 0.0020 | 0.0116 | 0.0012 |
| Schweinfurt (City) | 1* | 3 | 0.80 | 1.00 | 0.0254 | 0.0009 | 0.0014 | 0.0019 | 0.0152 | 0.0015 |
| Starnberg | 14 | 6 | 1.48 | 2.70 | 0.0212 | 0.0012 | 0.0008 | 0.0013 | 0.0113 | 0.0009 |
| Straubing-Bogen | 37 | 2 | 0.00 | 0.00 | 0.0195 | 0.0017 | 0.0008 | 0.0018 | 0.0147 | 0.0012 |
| Tirschenreuth | 26 | 3 | 0.57 | 1.70 | 0.0232 | 0.0023 | 0.0012 | 0.0023 | 0.0143 | 0.0010 |
| Traunstein | 35 | 1 | 0.60 | 0.60 | 0.0189 | 0.0010 | 0.0008 | 0.0022 | 0.0109 | 0.0014 |
| Weiden (Oberpfalz) | 1* | 1 | 2.90 | 2.90 | 0.0210 | 0.0032 | 0.0010 | 0.0018 | 0.0129 | 0.0010 |
| Weilheim-Schongau | 34 | 3 | 2.50 | 6.00 | 0.0195 | 0.0011 | 0.0010 | 0.0021 | 0.0126 | 0.0012 |
| W´burg-G´hausen | 27 | 13 | 10.7 | 24.0 | 0.0187 | 0.0012 | 0.0010 | 0.0018 | 0.0137 | 0.0010 |
| Wunsiedel (F´geb.) | 17 | 17 | 0.72 | 2.60 | 0.0255 | 0.0015 | 0.0014 | 0.0030 | 0.0152 | 0.0009 |
| Würzburg | 52 | 10 | 1.35 | 2.20 | 0.0215 | 0.0009 | 0.0007 | 0.0012 | 0.0106 | 0.0012 |
| Würzburg (City) | 1* | 2 | 1.95 | 2.90 | 0.0219 | 0.0006 | 0.0010 | 0.0014 | 0.0109 | 0.0013 |

*urban district

[1]no. of municipalities

[2]no. of U analyses
